# Characterization of the complete plastome of *Thalictrum aquilegiifolium* var. *sibiricum* (Ranunculaceae), an annual herb

**DOI:** 10.1080/23802359.2022.2087544

**Published:** 2022-07-25

**Authors:** Rui Wang, Bo-Qiang Tong, Xin-Yan Yang, Xue-Jie Zhang

**Affiliations:** a Shandong Provincial Key Laboratory of Plant Stress Research, College of Life Sciences, Shandong Normal University, Ji’nan, Shandong, China; b Shandong Provincial Center of Forest and Grass Germplasm Resources, Ji’nan, Shandong, China; c Campus Hospital, Taishan University, Tai’an, China

**Keywords:** *Thalictrum aquilegiifolium* var. *sibiricum*, plastome, phylogeny

## Abstract

*Thalictrum aquilegiifolium* var. *sibiricum*, is an annual herb that grows on slopes or in mountain gullies in areas of damp forest. In this study, we report for the first time the complete plastome sequence of *T. aquilegiifolium* var. *sibiricum*. The plastome sequence is 156,244 bp in length and comprises a large single-copy region (85,447 bp), a small single-copy region (17,599 bp), and a pair of inverted repeat regions (26,480 bp). The genome encodes 112 unique genes, including 78 protein-coding genes, 30 tRNAs, and four rRNAs, and has total GC content of 38.4%. Phylogenomic analysis based on the plastome sequences of 20 species in the family Ranunculaceae indicated that *T*. *aquilegiifolium* var. *sibiricum* is clustered with *Thalictrum minus*.

*Thalictrum aquilegiifolium* var. *sibiricum* (Linnaeus 1737) is a medicinal plant that is mainly distributed in China, Japan, and Korea (Flora of China, Chinese Academy of Sciences 2001). The genus *Thalictrum,* belonging to the subfamily Thalictroideae (Ranunculaceae), includes more than 200 species worldwide (Lv et al. 2020), among which 67 species are found in China, with 43 of these being known to have medicinal value (Chen et al. 2003). Plants in the genus *Thalictrum* are a rich source of alkaloids (Aynehchi 1979; Al-Howiriny et al. 2002), which have been shown to have a range of different pharmacological properties, including antitumor, antibacterial, and hypotensive activities. In addition to alkaloids, these plants also contain triterpenoids and flavonoids as active constituents (Lutskii et al. 2005; Khamidullina et al. 2006). In this study, we report for the first time the complete plastome sequence of *T. aquilegiifolium* var. *sibiricum*. The publication of this sequence will provide an important basis for phylogenetic research on the genus *Thalictrum* and make a significant contribution to the exploitation and utilization of its resources.

Fresh leaves of *T*. *aquilegiifolium* var. *sibiricum* were collected from Lushan National Forest Park (Shandong, China; 36°23′32.16″N, 118°6′10.01″E). The voucher specimen has been deposited at the College of Life Sciences, Shandong Normal University (Xue-Jie Zhang, zhangxuejie@sdnu.edu.cn) under the voucher number 091933B-2. Total genomic DNA was extracted from the leaf material using a modified CTAB method (Doyle and Doyle 1987; Guo et al. 2020) and used for library preparation and Paired-end (PE) sequencing performed using an Illumina Novaseq platform at Novogene (Beijing, China). The plastome sequence was assembled using getorganelle with the parameter –F set as embplant_pt (Jin et al. 2020). The read length was 150 bp and insert sizes were ca. 350 bp. The assembled plastome sequence was annotated using the Plastid Genome Annotator (PGA, https://github.com/quxiaojian/PGA) (Qu et al. 2019). To determine the phylogenetic position of *T*. *aquilegiifolium* var. *sibiricum*, we constructed a maximum-likelihood (ML) tree using RAxML v8.2.10 (Stamatakis 2014), which included a tree robustness assessment comprising 1000 rapid bootstrap replicates using the GTRGAMMA substitution model, based on an alignment of 78 shared protein-coding genes (PCGs) using MAFFT v7.313 (Katoh and Standley 2013).

The complete plastome of *T*. *aquilegiifolium* var. *Sibiricum* (GenBank accession number: MW816628) is 156,244 bp in length and comprises a large single-copy region (85,447 bp), a small single-copy region (17,599 bp), and a pair of inverted repeat regions (26,480 bp), with an overall GC content of 38.4%. We annotated a total of 112 unique genes in the plastome genome, including 78 PCGs, 30 tRNAs, and 4 rRNAs. Among these annotated genes, 13 of the PCGs and eight of the tRNAs contain introns. Phylogenomic analysis based on the plastome sequences of 20 species in the family Ranunculaceae indicated that *T*. *aquilegiifolium* var. *sibiricum* is clustered with *Thalictrum minus*. (Figure 1).

**Figure 1.**
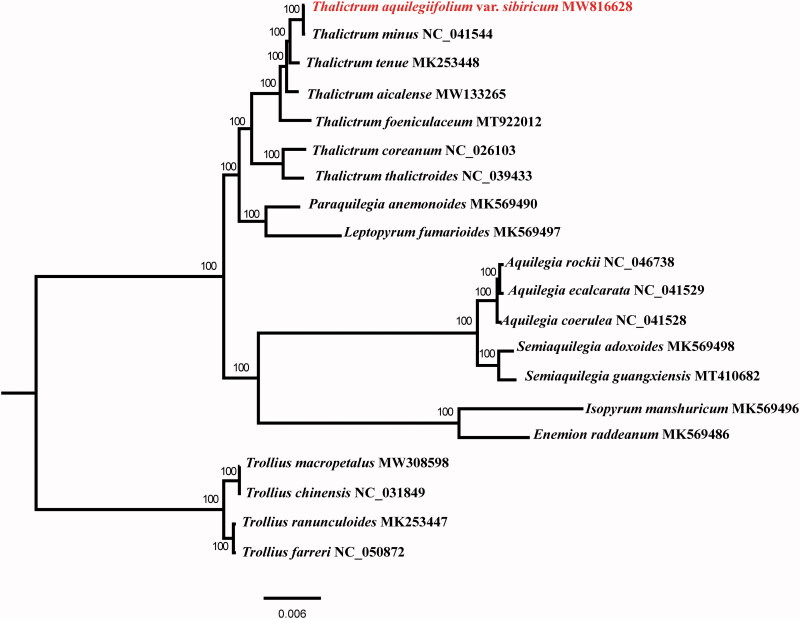
A maximum likelihood (ML) phylogenetic tree based on the plastome sequences of 20 species in the family Ranunculaceae. The numbers on branches are bootstrap support values.

## Ethical approval

No ethical approval required.

## Author contributions

Conceiving and designing, R.W., and X.-J.Z.; performing and analyzing data, X.-J.Z.; writing—original draft preparation, R.W.; writing—review and editing, B.-Q.T.; supervision, X.-Y.Y., B.-Q.T., and X.-J.Z.; All authors have read and agreed to the published version of the manuscript.

## Data Availability

The data that support the findings of this study are openly available in GenBank of NCBI at https://www.ncbi.nlm.nih.gov, reference number MW816628. The associated BioProject, SRA, and Bio-Sample numbers are PRJNA720285, SRR14161500, and SAMN18644861, respectively.
